# Chocolate, “Food of the Gods”: History, Science, and Human Health

**DOI:** 10.3390/ijerph16244960

**Published:** 2019-12-06

**Authors:** Maria Teresa Montagna, Giusy Diella, Francesco Triggiano, Giusy Rita Caponio, Osvalda De Giglio, Giuseppina Caggiano, Agostino Di Ciaula, Piero Portincasa

**Affiliations:** 1 Department of Biomedical Sciences and Human Oncology, Section of Hygiene, University of Bari Aldo Moro, Medical School, Piazza G. Cesare 11, 70124 Bari, Italy; giusy.diella@uniba.it (G.D.); francescotrigg@hotmail.it (F.T.); osvalda.degiglio@uniba.it (O.D.G.); giuseppina.caggiano@uniba.it (G.C.); 2 Department of Biomedical Sciences and Human Oncology, Clinica Medica “A. Murri”, University of Bari Aldo Moro, Medical School, Piazza G. Cesare 11, 70124 Bari, Italy; giusy.caponio@uniba.it (G.R.C.); agostinodiciaula@tiscali.it (A.D.C.); 3 Department of Soil, Plant and Food Science (DISSPA), University of Bari Aldo Moro, Via Amendola 165/a, 70126 Bari, Italy

**Keywords:** chocolate, cocoa, Food of the Gods, *Theobroma cacao*, nitric oxide, cardiovascular effects

## Abstract

Chocolate is well known for its fine flavor, and its history began in ancient times, when the Maya considered chocolate (a cocoa drink prepared with hot water) the “Food of the Gods”. The food industry produces many different types of chocolate: in recent years, dark chocolate, in particular, has gained great popularity. Interest in chocolate has grown, owing to its physiological and potential health effects, such as regulation of blood pressure, insulin levels, vascular functions, oxidation processes, prebiotic effects, glucose homeostasis, and lipid metabolism. However, further translational and epidemiologic studies are needed to confirm available results and to evaluate other possible effects related to the consumption of cocoa and chocolate, verifying in humans the effects hitherto demonstrated only in vitro, and suggesting how best to consume (in terms of dose, mode, and time) chocolate in the daily diet.

## 1. Background

The history of chocolate began with the Maya, who were probably the first people in South America to cultivate the cocoa plant [1]. For the Maya, chocolate was a cocoa drink prepared with hot water and often flavored with cinnamon and pepper. It was called the “Food of the Gods” and was presented at the table of Emperor Moctezuma II by the Aztecs [1].

In 1502, Christopher Columbus was the first European to encounter cocoa. He captured a canoe that contained cocoa beans, which were considered “mysterious-looking almonds” and identified as a form of currency in Mesoamerica [2,3].

Cocoa appeared in Europe in 1528, when the Spanish conquistador Hernán Cortés brought samples of cocoa to King Charles of Spain, spreading the great effects of the beverage prepared from this “brown gold” [3,4]. It was in 1753 that the Swedish scientist Carl Linnaeus named the cocoa plant *Theobroma cacao,* from the Latin name Theobroma [literally ‘food of the Gods’], and the Aztec word xocolatl [i.e., xococ (bitter) and atl (water)] [5].

The characteristics of chocolate were long ignored in Europe owing to difficulties with an environment unfavorable to its growth. The natural habitat of the cocoa tree is the lower level of an evergreen rain forest. Cocoa plants respond well to relatively high temperatures (with a maximum annual average of 30–32 °C and minimum average of 18–21 °C) and generally high relative humidity: often as much as 100% during the day, falling to 70–80% at night [6]. According to the latest published data of the International Cocoa Organization (ICCO), the total world production of cocoa beans in 2016–17 was 4,739,000 tons, principally from Africa (3,622,000 tons) [7]. 

Demand for organic cocoa products is also expanding, as consumers are increasingly concerned about food security and other environmental issues. However, the organic cocoa market still represents a very small share of the total cocoa market, estimated at less than 0.5% of total production [8].

In this review, we will discuss the main evidence relating to cocoa and chocolate, exploring the possible effects on human health related to their consumption.

## 2. Chocolate Varieties

**Figure 1:**
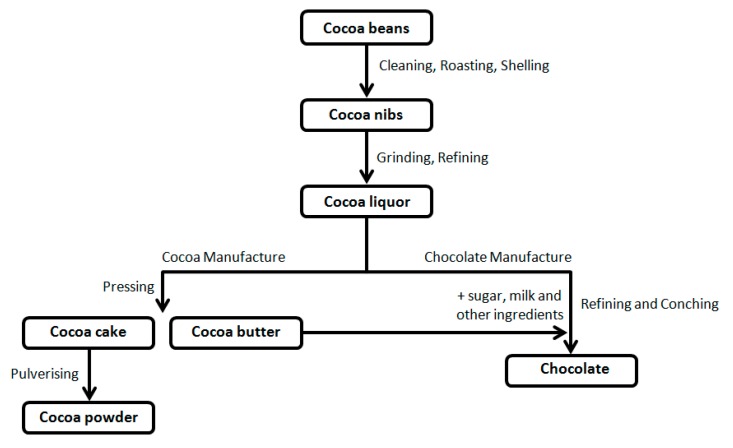
The processing of chocolate from cocoa beans.

Starting from cocoa beans, through various processes of transformation (Figure 1), the food industry produces different types of chocolate with defined ingredients and characteristics [1,9,10,11]. 

(1) Dark chocolate contains cocoa bean solids (up to 80% of the total weight) and cocoa butter. With the intense, persistent aroma of cocoa, it melts in the mouth, leaving a pleasant, bitter aftertaste. Its quality depends on the percentage of cocoa. Most of the health benefits attributable to chocolate are associated with consuming the dark type.

(2) Gianduja chocolate is a combination of hazelnuts, cocoa, and sugar; it is brown. 

(3) Milk chocolate contains cocoa butter, sugar, milk powder, lecithin, and cocoa (the latter not less than 20–25%). With a bright appearance, it has an intense, persistent aroma and sweet taste with a slightly bitter accent of cocoa.

(4) White chocolate contains cocoa butter, milk, and sugar with no cocoa solids; it has a sweet, pleasant taste. 

## 3. Nutritional Aspects

Cocoa, the basic ingredient in chocolate, contains a significant amount of fat (40–50% as cocoa butter, with approximately 33% oleic acid, 25% palmitic acid, and 33% stearic acid). It also contains polyphenols, which constitute about 10% of a whole bean’s dry weight [12]. Cocoa bean is one of the best-known sources of dietary polyphenols, containing more phenolic antioxidants than most foods [13]. Three groups of polyphenols can be identified in cocoa beans: catechins (37%), anthocyanidins (4%), and proanthocyanidins (58%); these flavonoids are the most abundant phytonutrients in cocoa beans [14,15,16]. However, the bitterness caused by polyphenols makes unprocessed cocoa beans rather unpalatable. Manufacturers have, therefore, developed processing techniques for eliminating the bitterness. Such processes decrease the polyphenol content by up to 10-fold: for consumers the product is markedly different, mainly owing to the low-polyphenol content [12,15] and the other substances added during the processing phase (e.g., sugar, emulsifiers such as soy lecithin). It is well known that polyphenols are associated with beneficial effects, therefore cocoa (rich in polyphenols) and dark chocolate (with a high percentage of cocoa and higher phenolic antioxidant compounds compared to the other chocolate varieties [13]) have assumed significant importance [17].

The nitrogenous compounds of cocoa include both proteins and methylxanthines (theobromine and caffeine) [18]. Cocoa is also rich in minerals: potassium, phosphorus, copper, iron, zinc, and magnesium [18]. The nutritional values of cocoa and two types of chocolate appear in Table 1 [13,19,20]. 

## 4. Lights and Shadows in Chocolate and Cocoa Consumption

Chocolate consumption has recently increased around the world; dark chocolate, in particular, has become very popular for its high concentrations of cocoa and beneficial effects on human health compared with normal or milk chocolate [21,22,23,24]. In addition, milk chocolate could be associated with adverse effects due to its sugar content.

Therefore, only dark chocolate, with high percentages of cocoa, flavonoids, and theobromine and low content of sugar, differently from milk chocolate or other types of chocolate, would be associated with health-promoting effects [11], including the prevention of cardiovascular disease. Similarly, cocoa induces positive effects on blood pressure, insulin resistance, and vascular function. It increases production of nitric oxide (NO) and has antioxidant effects, e.g., delayed oxidation of low-density lipoprotein (LDL) cholesterol and inhibiting ultraviolet-induced DNA oxidation [25,26]. 

The advantages and disadvantages of chocolate and cocoa consumption are discussed in the following sections, according to in vivo or in vitro studies. 

### 4.1. Cardiovascular Effects

A series of beneficial effects on the cardiovascular system might occur following regular intake of cocoa-containing foods and beverages. Benefits include effects on blood pressure, insulin resistance, and vascular and platelet function [25].

Polyphenols, abundant in cocoa and dark chocolate, activate endothelial NO synthase; that leads to generation of NO [27], which lowers blood pressure by promoting vasodilation [28,29,30,31,32,33]. Indeed, following the consumption of dark chocolate, effects include improvement of the pulse wave speed and of the atherosclerotic score index, with parietal relaxation of large arteries and dilation of small and medium-sized peripheral arteries. Higher concentrations of plasma epicatechins help release endothelium-derived vasodilators and increase the concentration of plasma procyanidins, which leads to greater NO production and bioavailability [32]. Once released, NO also activates the prostacyclin synthesis pathway, which acts as a vasodilator in synergy with NO, thereby contributing to thrombosis protection [17]. Further, the anti-inflammatory and vasoprotective properties of prostacyclin are enhanced by its ability to reduce plasma leukotrienes [17,34,35].

A meta-analysis of randomized trials report that both acute and chronic chocolate and cocoa ingestion effectively increased flow-mediated vasodilatation, reduced systolic and diastolic blood pressure, and reduced serum insulin levels [36]. In young and healthy adults, a daily ingestion of 20 g of higher cocoa chocolate (90%) for a 30-day period improved vascular function by reducing central brachial artery pressures and promoting vascular relaxation [37]. A Swedish prospective study linked chocolate consumption (≥3–4 servings/week) with lower risk of myocardial infarction and ischemic heart disease [38]. On the other hand, a large prospective study exploring data from 83,310 postmenopausal women free of pre-existing major chronic diseases found no association between chocolate consumption and risk of coronary heart disease, stroke, or both combined. Conversely, an increased risk existed among women less than 65 years, in the highest quintile of chocolate consumption [39]. A lack of association between chocolate intake and risk of atrial fibrillation was also reported in a large cohort of United States male physicians [40]. Another population-based, prospective study on 20,992 participants failed to demonstrate an association between high chocolate intake (up to 100 g/day) and incident heart failure [41]. A systematic review suggested that regular chocolate use (<100 g/week) may be linked with reduced cardiovascular risk, and that the most appropriate dose of chocolate consumption was 45 g/week, since higher levels might counteract the health benefits due to adverse effects linked with elevated sugar consumption [42]. These findings were similar to results from a large cohort of Swedish men, which showed a J-shaped association between chocolate consumption and incidence of heart failure, with protective effects absent in subjects consuming ≥1 serving per day [43].

Cocoa plays also a role in treating cerebral conditions, such as stroke; in fact, cocoa intake is associated with increased cerebral blood flow [44]. In the same way, daily chocolate consumption may reduce the likelihood of a stroke attack [18,45]. However, a large Japanese population-based, prospective cohort study reported an association between chocolate consumption and lower risk of stroke in women but not in men [26].

Table 2 shows the studies on cardiovascular effects related to cocoa or chocolate consumption.

### 4.2. Glucose Homeostasis

Cocoa components offer potential as antidiabetic agents, especially with type 2 diabetes mellitus (T2D). This aspect is of particular relevance owing to the emerging worldwide epidemic of metabolic syndrome, including obesity, T2D, and dyslipidemia [46].

Cocoa and flavonols improve glucose homeostasis by slowing carbohydrate digestion and absorption in the gut [47,48]. Indeed, cocoa extracts and procyanidins dose-dependently inhibit pancreatic α-amylase, pancreatic lipase, and secreted phospholipase A2 [48,49]. Cocoa and its flavonols improve insulin sensitivity by regulating glucose transport and insulin signaling proteins in insulin-sensitive tissues (liver, adipose tissue, and skeletal muscle) preventing in these tissues oxidative and inflammatory damage associated with the disease [47]. In younger and normal body-weight men, the results from the Physicians’ Health Study reported an inverse relation of chocolate consumption with incident diabetes [50]. In a multiethnic United States cohort, authors found a lower risk of developing T2D in subjects with the highest intake of chocolate products and cocoa-derived flavonoids [51]. A dose-response meta-analysis, however, suggested a nonlinear association between chocolate consumption and the risk of T2D, with a peak protective effect at 2 servings/week and no benefit recorded when increasing consumption was above 6 servings/week [52].

A prospective study in a large number of Japanese pregnant women also showed a lower risk of gestational diabetes in subjects in the highest quartile of chocolate consumption [53].

The observed effects on glucose homeostasis seem to be strongly dependent on the amount of polyphenols. In fact, a single-blind randomized placebo-controlled cross-over study showed, after 4 weeks, negative metabolic effects (i.e., raised fasting insulin, insulin resistance, and salivary cortisol) in subjects consuming 20 g/day dark chocolate with negligible polyphenol content but not in those consuming the same amount of polyphenol-rich (500 mg) chocolate [54].

Therefore, the daily consumption of small quantities of flavonols from cocoa or chocolate, associated with a dietary intake of flavonoids, would constitute a natural and economic approach to prevent or potentially contribute to the treatment of T2D with minimal toxicity and negative side effects [47]. However, most commercially available soluble cocoa products or chocolates contain low amount of flavonols and are rich in sugar and calories. Therefore, high consumption of chocolate will induce paradoxical consequences, i.e., weight gain and impaired glucose homeostasis, especially in T2D patients and obese individuals [48].

Table 3 shows the studies on glucose homeostasis effects related to cocoa or chocolate use.

### 4.3. Cancer

Results regarding the effects of cocoa/chocolate consumption on cancer are rather controversial. Early studies suggested that excess chocolate intake could be a predisposing factor to tumor development (as colorectal and breast cancer) [55,56].

According to other in vitro studies, cocoa inhibits the growth of cancer cells; however, the exact anticancer mechanisms are poorly understood [57,58]. 

Some authors demonstrated that cocoa liquor procyanidins significantly reduced the incidence and multiplicity of lung carcinomas and decreased thyroid adenomas developed in male rats, and inhibited mammary and pancreatic tumorigenesis in female rats [59,60]. Cocoa procyanidins also reduced vascular endothelial growth factor activity and angiogenic activity associated with tumor, determining down-regulation of tyrosine kinase ErbB2 [61]. 

In the last years, the treatment of different ovarian cancer cell lines with various concentrations of cocoa procyanidin-rich extract, inducing cytotoxicity and chemosensitization, showed a significant percentage of cells in sub-G1/G0 (hypodiploid) phase, which increased with increasing concentration, and a significant accumulation of cells in the S phase was seen [62]. This effect is probably due to an increase in intracellular levels of reactive oxygen species (ROS) [63]. In an animal study, a diet containing dark chocolate reduced the total number of aberrant crypt foci in the colon. The effect was associated with down-regulation in the transcription levels of both COX-2 and ReIA [64]. In addition, cocoa significantly decreased the tumor incidence and size in mice with colitis-associated cancer [65]. 

At present, further translational and prospective studies need to explore the intrinsic mechanisms of cocoa’s anticancer action to support its use as a co-adjuvant in preventing and treating cancer [18].

Table 4 shows the studies on cancer related to cocoa or chocolate use.

### 4.4. Obesity and Lipid Metabolism

Recently, some studies have investigated the preventive or therapeutic effects of cocoa and cocoa constituents against obesity and metabolic syndrome [66]. Administering cocoa to rats decreased visceral adipose tissue [67]. DNA analysis conducted on the liver and mesenteric fat tissue provided interesting clues. In that study, the authors observed decreased expression of various genes associated with fatty acid transport and synthesis in the liver and mesenteric fat as well as increased expression of genes associated with thermogenesis [18,67].

In a clinical study, smelling dark chocolate was assessed to evaluate an appetite response. Chocolate produced a satiation response and reduced appetite; thus, it could be helpful in preventing weight gain [68]. Further, flavonoids can produce metabolic events that induce reduction of lipogenesis, induction of lipolysis, and increased adiponectin secretion; such events reduce lipid deposition and insulin resistance, thus mitigating obesity [17].

A study reported a significantly greater and dose-dependent weight gain over time in subjects with more frequent chocolate consumption. However, no information was provided about the consumer profile of enrolled subjects and the type of chocolate consumed (in particular, the specific amount of dark chocolate) [69].

A recent meta-analysis reported the lack of effects of cocoa or dark chocolate on weight, body mass index (BMI), and waist circumference. However, a subgroup analysis showed reduced weight and BMI following cocoa/dark chocolate supplementation ≥ 30 g chocolate per day in trials between 4–8 weeks, pointing to the relevant role of the consumed dose and trial duration [70].

Dark chocolate might also operate in combination with other nutraceuticals, and have positive effects on lipid profile. Our group has recently reported distinct effects of 24 g almond varieties on organoleptic features and on gastrointestinal function (gallbladder and gastric emptying, orocecal transit) in healthy subjects [71]. One 4-week crossover feeding trial among 31 overweight or obese adults determined that daily consumption of almonds (42 g/day) alone or combined with dark chocolate was beneficial for total cholesterol, low-density (LDL) lipoprotein cholesterol, and apolipoprotein B concentrations. The authors concluded that incorporating almonds, dark chocolate, and cocoa into a diet without exceeding energy needs could reduce the risk of coronary heart disease [72]. 

A meta-analysis showed that, in the short term (2–12 weeks), dark chocolate/cocoa consumption can significantly lower total and LDL cholesterol levels, but has no effect on high-density lipoprotein HDL and triglycerides [73]. Similar results derive from a placebo-controlled cross-over study, in which daily consumption of cocoa flavonol-containing dark chocolate bars with added plant sterols significantly reduced serum total and LDL cholesterol [74].

Normal weight obese syndrome consists of an excessive body fat associated with a normal BMI, and a higher risk for cardiovascular morbidity and mortality. A group of normal weight obese women consuming dark chocolate (100 g/day, 70% cocoa) for a short period (one week) displayed a rise in the HDL cholesterol levels, and a decrease of the LDL/HDL cholesterol ratio and abdomen circumference. The authors concluded that the regular consumption of dark chocolate would help in maintaining a good atherogenic profile, due to the favorable effects on HDL cholesterol, lipoprotein ratios, and possibly on inflammation markers [75].

Table 5 shows the studies on obesity and lipid metabolism related to cocoa or chocolate use.

### 4.5. Intestinal Microbiota

In recent years, there is a growing interest in the study of intestinal microbiota and its changes as result of a particular diet. The human gut harvests the intestinal microbiota, a huge collection of microbes with a key role in energy storage and metabolic disorders [76]. Whereas flavonol monomers and dimers are absorbed in the small intestine, procyanidins undergo metabolization by colonic microbiota, with production of phenolic acids, subsequently absorbed, metabolized in the liver, and eliminated in the urine or in feces [77,78,79,80]. Thus, gut microbiota is responsible for the metabolization of polyphenols in other bio-active compounds (i.e., valerolactones [81], and various phenolic acids [82]) with potential anti-inflammatory properties [17].

A study conducted on rats fed with cocoa diet for 6 weeks highlighted a significant reduction of percent of *Bacteroides*, *Clostridium*, and *Staphylococcus*, changes of tool-like reception expression, and a reduction of immunoglobulin A intestinal secretion, significantly correlated with the decrease in the proportion of the *Clostridium* and *Streptococcus* [78].

In pigs, cocoa consumption, in addition to determining changes in metabolites in biofluids and tissues, as the increase in O-methyl-epicatechin glucuronide conjugates in serum, urine, and visceral adipose tissue, induced a significant increase of the abundance of *Lactobacillus* species from the *casei* group in feces and *Bifidobacterium* species in proximal colon contents [83]. 

Tzounis et al. [79] conducted the first human-intervention study designed to investigate the influence of high cocoa flavanol intake on the growth of the human fecal microbiota. In particular, these authors assessed that the intake of 494 mg of cocoa flavonoids/ day for 4 weeks had a significant effect on intestinal microbiota growth.

Table 6 shows the studies on intestinal microbiota related to cocoa or chocolate use.

### 4.6. Immune System

In vivo and in vitro studies showed that cocoa has regulatory properties on the immune cells implicated in both innate and acquired immunity. In animals, these effects are present at systemic and intestinal level [84,85]. In Lewis rats a 10% cocoa diet or a 0.25% theobromine diet were both able, after one week, to lower serum concentrations of IgG, IgM, IgA, and intestinal IgA, as compared with control diet. Both cocoa and theobromine modified the thymocyte composition increasing CD4-CD8- and CD4+CD8- proportions, and changed the composition of mesenteric lymph node (reduced percentage of T-helper) and spleen (increased proportion of T-helper). Taken together, the data suggest that theobromine is the agent mediating the major immunoregulatory effects of cocoa [86]. Dark chocolate consumption was found having anti-inflammatory effects in a 4-week randomized clinical trial, which was especially visible in the reduced post-challenge responses of cytokines, vascular markers, white blood cells, and leukocyte-activation markers [87,88].

Regular cocoa consumption could be related to preventing or improving health imbalance induced by allergic processes [89]. The positive effects of cocoa flavonoids on the immune system (related to several allergic mechanisms) are known, such as reducing the release of mediators, restoring the balance of T-helper 1 and T-helper 2 cells [90], and down-regulation of IgE production [89,91]. By contrast, chocolate is one of the main potentially allergenic foods that is also capable of causing hypersensitivity reactions, manifesting different clinical symptoms (e.g., fatigue, irritability, insomnia, headache, asthma, and diarrhea) which appear in a few hours or days after food intake [92]. 

Table 7 shows the studies on the immune system related to cocoa or chocolate use.

### 4.7. Central Nervous System

There is evidences of some beneficial effects on the central nervous system, but larger, prospective studies are missing, so far.

In healthy volunteers, the ingestion of 100 g dark chocolate (72% cocoa) increased [^18^F] fluorodeoxyglucose (^18^F-FDG) uptake in the visual cortex, in somatosensory, motor, and pre-frontal cortices, as shown by combined positron emission tomography-computed tomography (PET-CT) [22]. These findings point to dark chocolate-dependent acute effects on cerebral function [22]. The polyphenols in dark chocolate could act on the central nervous system (CNS) and neurological functions through the production of NO [11,17]. Vasodilation and increased cerebral blood flow provide oxygen and glucose to neurons, leading to increased formation of blood vessels in the hippocampus [11,93]. The polyphenol-dependent antioxidant potential could contribute to amelioration of some neurodegenerative disorders [11,93,94]. This inference is based on the fact that age-related cognitive impairment and disorders, such as Alzheimer’s and Parkinson’s diseases, are related to the accumulation of reactive oxygen species in the brain [11,94,95]. 

The effect of cocoa bioactives on signaling pathways in neurocytes may provide another support for linking dark chocolate with regulation of brain function [11]. Cocoa flavonols and methylxanthines can activate the cascade pathways of such molecules as rapamycin that play a crucial role in synaptic function, neuronal growth, memory mechanisms, and the pathogenesis of neurodegenerative disorders [96].

A prospective study on elderly subjects (age ≥65 years) with normal mini-mental state examination at entry showed that chocolate intake was linked with a decreased risk of cognitive decline during a median follow up of 48 months [97]. Results from a cross-sectional analysis in subjects aged 23–98 years showed a better cognitive performance in those consuming chocolate more frequently. However, following a prospective observation, a relationship between cognitive function and chocolate intake was not confirmed when measured up to 18 years later [98].

### 4.8. Psychological Aspects

The social and psychological context of everyday life affects metabolic health, emotions, and moods; it can play a role in determining dietary choices [99,100]. In some cases, chocolate consumption can be indirectly associated with a form of depression: hysteroid dysphoria. This condition involves frequent episodes of depression in response to feeling inadequate or socially rejected, which culminates in true bulimic attacks for confectionery and chocolate. A true chocolate addiction (being chocoholic) is akin to alcoholism and nicotine dependence; it affects 40% of the female and 15% of the male population in Western countries [101]. The symptoms involve being responsive to drugs that enhance serotonin transmission; this suggests that central serotonin pathways may be involved in chocolate consumption. The presence of serotonin could explain why sugar and confectionery are strongly desired during chocolate bulimic crises. The ingestion of carbohydrates (e.g., bread and chocolate) increases the relationship between plasma tryptophan and other neutral amino acids; consequently, the transport of tryptophan through the blood–brain barrier is activated, with an increase in cerebral serotonin synthesis, which produces a feeling of energy and pleasure [102]. 

### 4.9. Sexual Aspects

Chocolate exerts several effects on human sexuality, mainly acting as an aphrodisiac [103]. Cocoa powder and chocolate contain three unsaturated N-acylethanolamines, which, acting as cannabinoid mimics, could activate cannabinoid receptors or increase anandamide concentrations [103,104]. The latter, in conjunction with other components of chocolate (such as caffeine and theobromine), produces a transient feeling of well-being. Anandamide enhances sexual performance in male rats [103,105]. Moreover, serotonin has been found in several regions of the female genital tract in humans and other animals, where it acts on vasoconstriction and vasodilatation. The principal component of sexual arousal is peripheral vasocongestion of genital tissues; thus, serotonin could be involved in the process of sexual stimulation [103].

Table 8 shows the studies on the nervous system, and psychological and sexual aspects related to cocoa or chocolate use.

## 5. Conclusions

Cocoa and chocolate act as functional foods, since both carry a number of substances contributing to beneficial health effects. Chocolate combines some organoleptic characteristics with aphrodisiac and antidepressant properties, extending its effects beyond the cardiovascular system, metabolic diseases, CNS diseases, and psychological profiles.

We should stress that several studies evaluated the health-promoting properties of cocoa and not of chocolate itself.

Moreover, because in chocolate processing, cocoa loses some of the polyphenol compounds (the main constituents responsible for the beneficial effects on health), we think that the role of chocolate on human health cannot be completely compared to that of cocoa. Despite the availability of a number of in vitro and experimental reports, epidemiological studies assessing possible beneficial effects of chocolate (in particular dark chocolate) are still scarce. One should keep in mind the presence of a number of confounders (i.e., other diet components, lifestyle, environmental exposures, exact consumption of chocolate, chocolate composition, duration of observation, and other risk factors). Such conditions strongly limit the strength of evidences.

In conclusion, further translational studies need to evaluate all possible effects related to consuming chocolate and to verify in humans the effects hitherto demonstrated only in vitro and on animals. This approach could suggest how best to consume (in terms of dose, mode, and time) chocolate in the daily diet, considering eating habits and lifestyle.

## Figures and Tables

**Table 1:** Nutritional values per 100 g of cocoa and two types of chocolate.

Chemical Composition	Cocoa	Dark Chocolate	Milk Chocolate
Water (g)	2.5	0.5	0.8
Protein (g)	20.4	6.6	7.3
Lipid (g)	25.6	33.6	36.3
Cholesterol (mg)	0	0	10
Carbohydrate (g)	11.5	49.7	50.5
Sugar (g)	traces	49.7	50.5
Total fiber (g)	-	8	3.2
Sodium (mg)	-	11	120
Potassium (mg)	-	300	420
Iron (mg)	14.3	5	3
Calcium (mg)	51	51	262
Phosphorus (mg)	685	186	207
Thiamin (mg)	0.08	0.07	0.09
Riboflavin (mg)	0.3	0.07	0.39
Niacin (mg)	1.7	0.6	0.6
Vitamin A (µg)	7	9	25
Phenolics (mg)	996–3781	579	160
Flavonids (mg)	-	28	13
Theobromine (mg)	-	802	125
Energy (kcal)	355	515	545
Energy (kJ)	1486	2155	2281

**Table 2:** Studies on cardiovascular effects related to cocoa or chocolate consumption, included in this review.

Study	Study Design	Food Type	Main Outcomes
Dong J-Y. et al. 2017 [26]	Prospective human cohort study	Chocolate	Inverse association between chocolate consumption and risk of developing stroke in women
Engler M.B. et al. 2004 [29]	Randomized controlled trial in human	Chocolate	Dark chocolate improved endothelial function and increased concentration of plasmatic epicatechins in healthy adults
Fisher N.D. & Hollenberg N.K. 2006 [30]	Randomized controlled trial in human	Cocoa	Cocoa enhanced several measures of endothelial function (nitric oxide-dependent) to a greater degree among older, in whom endothelial function is more disturbed, than younger healthy subjects
Fisher N.D. et al. 2003 [31]	Randomized controlled trial in human	Cocoa	Cocoa induced vasodilation via activation of the nitric oxide system, providing a plausible mechanism for the protection that flavanol-rich foods induce against coronary events
Murphy K.J. et al. 2003 [33]	Randomized, double-blind, placebo-controlled study	Cocoa	Cocoa flavanol and procyanidin supplementation significantly increased plasma epicatechin and catechin concentrations and significantly decreased platelet function
Schramm D.D. et al. 2003 [34]	Randomized controlled trial in human	Cocoa	Valuating the food effects on the absorption and pharmacokinetics of cocoa flavanols, carbohydrates increased oral flavanol absorption
Schwab U.S. et al. 1996 [35]	Randomized crossover trial in human	Cocoa	Palmitic acid-enriched diet (using palm oil) increased serum lipids, lipoproteins and plasma cholesteryl ester transfer protein activity compared with the stearic acid-enriched diet (using cocoa butter)
Pereira T. et al. 2019 [37]	Randomized double-blind trial in human	Chocolate	Cocoa-rich chocolate improved vascular function by reducing central brachial artery pressures and promoting vascular relaxation in young, healthy adults
Larsson S.C. et al. 2016 [38]	Prospective human study	Chocolate	Chocolate consumption was associated with lower risk of myocardial infarction and ischemic heart disease
Greenberg J.A. et al. 2018 [39]	Prospective human study	Chocolate	No association between chocolate intake and risk of coronary heart disease, stroke, or both combined was observed
Khawaja O. et al. 2015 [40]	Randomized double-blind controlled human study	Chocolate	No support to association between chocolate consumption and risk of atrial fibrillation among male physicians
Kwok C.S. et al. 2016 [41]	Prospective human study	Chocolate	Habitual chocolate consumption was not associated with the risk of incident heart failure among healthy men and women
Steinhaus D.A. et al. 2017 [43]	Prospective cohort human study	Chocolate	J-shaped relationship between chocolate consumption and heart failure incidence
Francis S.T. et al. 2006 [44]	Randomized controlled trial in human	Cocoa	Measurements of arterial spin labeling cerebral blood flow demonstrated an increase in blood flow after ingestion of flavanol-rich cocoa, suggesting its potential use for treatment of vascular impairment
Walters M.R. et al. 2013 [45]	Randomized controlled trial in human	Chocolate	Chocolate consumption is associated with an acute change in cerebral vasomotor reactivity, independent of metabolic and hemodynamic parameters.

**Table 3:** Studies on glucose homeostasis effects related to cocoa or chocolate use, included in this review.

Study	Study Design	Food Type	Main Outcomes
Gu Y. et al. 2011 [49]	In vitro porcine study	Cocoa	Cocoa extracts and cocoa procyanidins inhibited enzymes for digestion of carbohydrates and lipids, suggesting a role in body weight management in conjunction with a low calorie diet
Matsumoto C. et al. 2015 [50]	Randomized human study	Chocolate	Inverse relation of chocolate intake with incident diabetes mellitus in younger and normal–body weight men
Maskarinec G. et al. 2019 [51]	Cohort human study	Chocolate products	Participants with higher chocolate consumption and higher flavanol intake from cocoa products experienced a lower risk of developing type-2 diabetes
Yuan S. et al. 2017 [52]	Prospective human study	Chocolate	Chocolate consumption was associated with decreased risks of coronary heart disease, stroke, and diabetes
Dong J-Y et al. 2019 [53]	Prospective cohort human study	Chocolate	Chocolate consumption was associated with a lower risk of gestational diabetes mellitus
Almoosawi S. et al. 2012 [54]	Single-blind randomized placebo-controlled cross-over human study	Chocolate	Metabolic benefits of consuming polyphenol-rich dark chocolate and possibility of adverse effects occurring with polyphenol-poor chocolate

**Table 4:** Studies on cancer related to cocoa or chocolate use, included in this review.

Study	Study Design	Food Type	Main Outcomes
Boutron-Ruault M.C. et al. 1999 [55]	Randomized controlled trial in human	Chocolate	Chocolate intake resulted a risk factor to colorectal tumor development
Carnesecchi S. et al. 2002 [57]	In vitro human study	Cocoa	Cocoa polyphenols interfered with polyamine metabolism, showing an important anti-proliferative effects
Yamagishi M. et al. 2002 [59]	In vitro and in vivo rat study	Cocoa	Cocoa liquor proanthocyanidins inhibited mutagenicity of 2-amino-1-methyl-6-phenylimidazo[4,5-b]pyridine (PhIP) and rat pancreatic carcinogenesis in the initiation stage, but not mammary carcinogenesis induced by PhIP
Yamagishi M. et al. 2003 [60]	In vivo rat study	Cocoa	Cocoa liquor proanthocyanidins exerted chemopreventive effects in the lung, decreasing the incidence and multiplicity of carcinomas, and the quantitative values of adenomas in a dose-dependent manner in the thyroid
Kenny T. et al. 2004 [61]	In vitro human study	Cocoa	Down-regulation of tyrosine kinase ErbB2 and inhibition of human aortic endothelial cell growth by cocoa procyanidins
Taparia S. & Khanna A. 2016 [62]	In vitro human study	Cocoa	Treatment of ovarian cancer cell lines with cocoa procyanidin-rich extract showed a significant percentage of cells in sub-G1/G0 phase and a significant accumulation of cells in the S phase
Taparia S.S. & Khanna A. 2016 [63]	In vitro human study	Cocoa	Procyanidin-rich extract of natural cocoa powder caused ROS-mediated caspase-3 dependent apoptosis and reduction of pro-MMP-2 in epithelial ovarian carcinoma cell lines
Hong M.Y. et al. 2013 [64]	In vitro rat study	Chocolate	Chocolate diet-fed animals downregulated transcription levels of COX-2 and RelA and lowered the proliferation index
Saadatdoust Z. et al. 2015 [65]	In vitro mice study	Cocoa	Cocoa diet suppresses colitis-associated cancer tumorigenesis

**Table 5:** Studies on obesity and lipid metabolism related to cocoa or chocolate use, included in this review.

Study	Study Design	Food Type	Main Outcomes
Gu Y. et al. 2014 [66]	In vitro mice study	Cocoa	Dietary supplementation with cocoa in obese mice ameliorates obesity-related inflammation, insulin resistance, and fatty liver disease
Matsui N. et al. 2005 [67]	In vivo rat study	Cocoa	Cocoa ingestion decreased fatty acid synthesis and transport in liver and white adipose tissues, determining a body weight, mesenteric white adipose tissue weight and serum triacylglycerol concentrations lower in rats fed the cocoa diet than in those fed the mimetic cocoa diet
Massolt E.T. et al. 2010 [68]	Randomized controlled trial in human	Chocolate	Smell or ingestion of dark chocolate determined suppression of appetite because of the changes in ghrelin.
Greenberg J.A. et al. 2013 [69]	Prospective human cohort study	Chocolate	Habitual chocolate consumption was associated with long-term weight gain, in a dose-response manner
Lee Y. et al. 2017 [72]	Randomized controlled trial in human	Chocolate and cocoa	Consumption of almonds alone or combined with dark chocolate under controlled-feeding conditions improved lipid profiles
Allen R.R. et al. 2008 [74]	Double-blind placebo-controlled cross-over human study	Chocolate	Regular consumption of chocolate bars containing plant sterols and cocoa flavanols as part of a low-fat diet supported cardiovascular health by lowering cholesterol and improving blood pressure
Di Renzo L. et al. 2013 [75]	Case-control human study	Chocolate	Regular consumption of dark chocolate determined favourable effects on HDL cholesterol, lipoprotein ratios and inflammation markers in normal weight obese women

**Table 6:** Studies on intestinal microbiota related to cocoa or chocolate use, included in this review.

Study	Study Design	Food Type	Main Outcomes
Wiese S. et al. 2015 [77]	Randomized, double-blind, cross-over human study	Cocoa	Comparative biokinetics and metabolism of pure monomeric, dimeric, and polymeric flavan-3-ols
Massot-Cladera M. et al. 2012 [78]	In vivo rat study	Cocoa	Cocoa intake affected the growth of certain species of gut microbiota in rats and changes in the toll-like receptor pattern and in the intestinal immune system
Tzounis X. et al. 2011 [79]	Randomized controlled double-blind crossover trial in human	Cocoa	Consumption of the high–cocoa flavanol drink modified the gut microflora, reducing the plasmatic triacylglycerol and C-reactive protein concentrations.
Urpi-Sarda M. et al. 2007 [82]	In vivo human and rat study	Cocoa	Sensitivity and recovery of epicatechin, procyanidins, and phenolic microbial metabolites after cocoa intake in human and rat urine
Jang S. et al. 2016 [83]	In vivo and in vitro pig study	Cocoa	Consumption of cocoa powder enhanced the abundance of *Lactobacillus* and *Bifidobacterium* species and induced a reduction of tumor necrosis factor-α and toll-like receptor gene expression in intestinal tissues

**Table 7:** Studies on immune system effects related to cocoa or chocolate use, included in this review.

Study	Study Design	Food Type	Main Outcomes
Ramiro-Puig E. et al. 2008 [85]	In vivo and in vitro rat study	Cocoa	Cocoa-enriched diet modulated intestinal immune responses in young rats
Camps-Bossacoma M. et al. 2018 [86]	In vivo and in vitro rat study	Cocoa	Theobromine in cocoa was responsible for systemic and intestinal antibody concentrations and for modifying lymphocyte composition in young healthy rats
Esser D. et al. 2014 [87]	Randomized double blind crossover human study	Chocolate	Dark chocolate consumption improved leukocyte adhesion factors and vascular function in overweight men
Rodríguez-Lagunas M.J. et al. 2019 [89]	Cross-sectional observational human study	Cocoa	Consumption of cocoa was inversely correlated with physical activity and allergies. Moderate cocoa consumers had less frequency of chronic disease than the low consumers
Abril-Gil M. et al. 2012 [91]	In vivo rat study	Cocoa	Cocoa-enriched diet produced an immunomodulatory effect that prevented anti-allergen IgE synthesis

**Table 8:** Studies on the nervous system, and psychological and sexual aspects related to cocoa or chocolate use, included in this review.

Study	Study Design	Food Type	Main Outcomes
Fox M. et al. 2019 [22]	Randomized controlled trial human study	Chocolate	Dark chocolate with a high cocoa content has effects on colonic and cerebral function in healthy volunteers
Madhavadas S. et al. 2016 [94]	In vivo and in vitro rat study	Chocolate	Dark chocolate enhanced cognitive function and cholinergic activity in the hippocampus and corrected metabolic disturbances of rats
Moreira A. et al. 2016 [97]	Prospective cohort human study	Chocolate	Chocolate intake was associated with a lower risk of cognitive decline
Chrichton G.E. et al. 2016 [98]	Longitudinal human study	Chocolate	Chocolate intake was associated with better cognitive function
Martin F.I. et al. 2012 [100]	Randomized controlled trial in human	Chocolate	Snacks differing in sensory properties and presentation differently influenced postprandial anxiety, energy and emotional states
Salonia A. et al. 2006 [103]	Observational human study	Chocolate	Positive association between daily chocolate intake and sexual function.
